# Oral Symptoms Associated with COVID-19 and Their Pathogenic Mechanisms: A Literature Review

**DOI:** 10.3390/dj9030032

**Published:** 2021-03-11

**Authors:** Hironori Tsuchiya

**Affiliations:** School of Dentistry, Asahi University, Mizuho, Gifu 501-0296, Japan; tsuchi-hiroki16@dent.asahi-u.ac.jp; Tel.: +81-58-329-1263

**Keywords:** COVID-19, oral symptom, prevalence, pathogenic mechanism, gustatory dysfunction, xerostomia, mucosal lesion, SARS-CoV-2

## Abstract

Since the worldwide spread of severe acute respiratory syndrome coronavirus 2 (SARS-CoV-2) infection, management of coronavirus disease 2019 (COVID-19) has been challenging for healthcare providers. The oral cavity is referred to as a target of SARS-CoV-2. The aim of this study was to review oral symptoms specific to COVID-19 patients from the point of view of symptom prevalence and pathogenesis and to speculate the pathogenic mechanisms underlying them. Scientific articles were retrieved by searching PubMed/MEDLINE, Google Scholar, medRxiv, and bioRxiv from 3 February 2020 to 31 December 2020, and they were reviewed by title, abstract, and text for relevance. The literature search indicated that COVID-19 patients frequently present with gustatory dysfunction, xerostomia, and oral mucosal lesions, while their prevalence is likely to vary by country, age, gender, and disease severity. Gustatory dysfunction and xerostomia appear at an early stage of SARS-CoV-2 infection and last relatively long. One of possible pathogenic mechanisms of both symptoms is attributed to the expression of viral cellular entry-relevant receptors in taste cells and salivary glands. Dental professionals who can first become aware of oral symptoms associated with COVID-19 will play a more active role in and make a greater contribution to diagnosis and prevention of COVID-19.

## 1. Introduction

Highly infectious pneumonia first emerged in Wuhan, China in late 2019, and since then, the causative novel coronavirus or severe acute respiratory syndrome coronavirus 2 (SARS-CoV-2) quickly spread worldwide, causing a global pandemic of coronavirus disease 2019 (COVID-19) [1]. Management (early diagnosis and preventive measures) of COVID-19 has been challenging for healthcare providers, including dental professionals. Fever, cough, dyspnea, myalgia, and fatigue are among the most commonly reported manifestations, followed by headache, diarrhea, sputum, and hemoptysis [2,3]. However, a considerable number of COVID-19 patients remain asymptomatic or have mild symptoms, requiring a predictive index alternative or additional to these manifestations.

The process of SARS-CoV-2 entering target cells is mediated by binding to cellular angiotensin-converting enzyme 2 (ACE2) receptor and the subsequent priming of viral spike proteins by transmembrane protease serine 2 (TMPRSS2). ACE2 and TMPRSS2 are expressed in epithelial cells of human tongue and gingiva, especially enriched in dorsal tongue [4] and localized in fungiform papillae taste cells [5]. ACE2 and TMPRSS2 are also expressed in submandibular, parotid, and minor salivary glands [6], suggesting that salivary glands may be a potential reservoir for asymptomatic infection [7] and release viral particles via salivary ducts [8]. In fact, SARS-CoV-2 is consistently detected in saliva of COVID-19 patients with high viral loads [9,10]. Given the specific expression of ACE2 and TMPRSS2 in taste cells and salivary glands, SARS-CoV-2 infection is presumed to affect gustatory function and saliva secretion [11,12]. The oral cavity susceptible to SARS-CoV-2 is considered to not only provide the potential site of human-to-human viral transmission but also present symptoms characterizing COVID-19 [13,14,15]. In addition, COVID-19 may induce oral mucosal ulceration and inflammation because SARS-CoV-2 has the mucotropic activity and the property to dysregulate immune system and trigger cytokine storm [16,17,18].

ACE2 and TMPRSS2 are also highly expressed in human olfactory epithelia as well as in the oral cavity [19,20]. Since SARS-CoV-2 targets the olfactory system for entering host cells, the sense of smell is very likely to be affected in COVID-19 patients. In fact, chemosensory (gustatory and olfactory) disorders have been frequently reported by pre-symptomatic or asymptomatic patients. Taste alteration is more prevalent than smell alteration in a considerable number of studies [21,22,23]. However, the relevant studies thus far tend to lean toward olfactory dysfunction rather than gustatory dysfunction or to not distinguish between gustatory and olfactory symptoms [24,25,26]. In addition, the detailed pathogenic mechanisms of the oral symptoms remain unclear. If a study is conducted to elucidate them, one would expect that the outcome contributes to diagnosis and preventive measures of COVID-19.

From the point of view of symptom prevalence and pathogenesis, the present literature review focused on oral symptoms associated with CODIV-19 to characterize gustatory dysfunction, xerostomia, and mucosal lesions. The pathogenic mechanisms underlying these oral symptoms were also speculated.

## 2. Materials and Methods

Scientific articles were retrieved by searching PubMed/Medline, Google Scholar, medRxiv, and bioRxiv from 3 February 2020 to 31 December 2020. The publications earlier than 2020 were cited when they are essential to advancing the discussion. A literature search was carried out by using the following terms or combinations thereof: “COVID-19”, “SARS-CoV-2”, “oral symptom”, “gustatory dysfunction”, “gustatory disorder”, “taste impairment”, “dysgeusia”, “hypogeusia”, “ageusia”, “taste loss”, “xerostomia”, “dry mouth”, “hyposalivation”, “ulcer”, “stomatitis”, “halitosis”, “aphthous”, “sialadenitis”, “parotitis”, and “periodontal disease”. A literature review was limited to papers published in English. Studies lacking demographic characteristics and prevalence assessments for gustatory dysfunction were excluded. References of the retrieved articles were also searched for additional references. Collected articles were reviewed by title, abstract, and text for relevance.

## 3. Gustatory Dysfunction

Results of the literature search indicated that gustatory dysfunction is frequently present in COVID-19 patients diagnosed by reverse transcription-polymerase chain reaction (RT-PCR) test and serological antibody test. Table 1 summarizes the relevant studies.

**Table 1:** Gustatory dysfunction associated with coronavirus disease 2019 (COVID-19).

Subjects	Severity of Disease	Country or Ethnicity	Number of Patients	Mean or Median Age (Range)	Male (%)	Assessment Method	Symptom	Prevalence (%)	Reference
HP diagnosed according to the official guideline and by SARS-CoV-2 nucleic acid detection	Mild to critical	China	108	52.0 (one patient excluded)	48.1	Self-reported questionnaire	Amblygeusia	Total: 47.2 Male: 36.5 Female: 57.1	Chen et al. [9] *
HP diagnosed by RT-PCR test	Mild to moderate	12 European hospitals (European 93.3%, African 3.6%, South American 2.7%, North American 0.2%, Asian 0.2%)	417	36.9 (19–77)	36.9	Self-reported questionnaire about salty, sweet, bitter and sour taste modalities	Gustatory dysfunction characterized by impairments of four tastes	88.8	Lechien et al. [22] *
HP diagnosed by real-time RT-PCR test	Mild to severe	Iran	60	46.6	66.7	Validated UPSIT	Taste loss Taste and smell loss	6.7 16.7	Moein et al. [24]
HP (diagnostic method, NS)	NS	Italy	59	60 (50–74)	67.8	Self-reported questionnaire	Gustatory dysfunction Dysgeusia Ageusia	28.8 8.5 1.7	Giacomelli et al. [27] *
Patients diagnosed by RT-PCR test	Mild (93.1%) Moderate to severe (6.9%)	USA	59	(18–79)	49.2	Self-reported online questionnaire	Ageusia	71.2	Yan et al. [28]
Patients diagnosed by RT-PCR test	Mild to moderate	Subscribers of COVID-19 symptom tracker application of UK	579	40.8	30.9	Self-reported questionnaire	Ageusia and anosmia	59.4	Menni et al. [29] ^#^
HP diagnosed by real-time RT-PCR test	Non-severe (58.9%) Severe (41.1%)	China	214	52.7	40.7	Retrospective symptom survey	Taste impairment	5.6	Mao et al. [30] *
Patients diagnosed presumably by RT-PCR test	Mild to moderate	Italy	72	49.2 (26–90)	37.5	Objective evaluation using four tastants	Mild hypogeusia Moderate hypogeusia Severe hypogeusia Ageusia	22.2 15.3 9.7 1.4	Vaira et al. [31] *
HQP diagnosed by RT-PCR test	Mild	Italy	202	56 (45–67)	48.0	Self-reported questionnaire	Taste/smell alteration Very mild Mild or light Moderate Severe	64.4 2.5 11.4 13.4 13.4	Spinato et al. [32] *
HP diagnosed by RT-PCR test	Mild	Israel	42	34 (15–82)	54.8	Self-reported questionnaire	Dysgeusia	33.3	Levinson et al. [33] *
Patients diagnosed by real-time RT-PCR test	NS	Italy	72	49.7 (19–70)	54.2	Self-report of symptoms	Gustatory dysfunction Dysgeusia	72.2 25.0	Gelardi et al. [34]
OP diagnosed by RT-PCR test or clinical features	NS	France	1487	44 (32–57)	47.1 (35 excluded)	Observational survey	Gustatory dysfunction Ageusia	50.9 28.4	Lapostolle et al. [35]
Patients diagnosed by RT-PCR test	NS	France	68	NS	NS	Self-reported questionnaire	Hypogeusia	61.8	Bénézit et al. [36]
IP (37%) and OP (63%) diagnosed by real-time RT-PCR	Mild to severe	France	54	47	33.3	Self-reported symptoms	Dysgeusia	85.2	Klopfenst et al. [37]
HP diagnosed by RT-PCR test	Non-severe to severe	Spain	79	61.6	60.8	Self-reported questionnaire	Ageusia Hypogeusia Dysgeusia	17.7 8.9 10.1	Beltrán- Corbellini et al. [38]
HP diagnosed by real-time RT-PCR test	Severe and others	USA	16	65.5	75.0	Self-reported questionnaire	Dysgeusia	18.8	Aggarwal et al. [39]
HP diagnosed by real-time RT-PCR test	Mild to severe	Singapore	154	NS	NS	Self-reported questionnaire	Gustatory or olfactory dysfunction	22.7	Wee et al. [40]
OP and IP diagnosed by RT-PCR test	Mild (79.7%) Severe (20.3%)	USA (White 30.8%, Black 11.5%, Hispanic 26.9%, Asian 15.4%, Others 15.4%)	OP: 102 IP: 26	OP: 43 (34–54) IP: 53.5 (40–65)	OP: 51 IP: 34.6	Self-reported taste and smell sense	Dysgeusia	OP: 62.7 IP: 23.1	Yan, et al. [41]
HQP diagnosed presumably by RT-PCR test	Asymptomatic (27.3%) Mild (45.4%) Moderate (27.3%)	Italy	33	47.2 (26–64)	33.3	Self- or operator- administered test using four tastants	Mild hypogeusia Moderate hypogeusia Severe hypogeusia Ageusia	24.2 12.1 9.1 6.1	Vaira et al. [42] *
Patients diagnosed by real-time RT-PCR test	Mild	South Korea	172	26 (22–47)	38.4	Self-reported questionnaire	Hypogeusia	33.7	Kim et al. [43]
OP diagnosed by RT-PCR test	NS	Germany	72	38 (21–87)	56.9	Self-reported questionnaire	Gustatory dysfunction	69.4	Luers et al. [44]
Patients (diagnostic method, NS)	All stages Asymptomatic to mild (73.4%) Moderate (10.6%) Severe (2.2%) Critical (1.3%)	South Korea	3191 2342 339 71 41	44 (25–58)	36.4	Telephone interview	Ageusia	11.1	Lee et al. [45] *
Patients diagnosed by RT-PCR test	NS	Switzerland	103	46.8	48.5	Self-reported questionnaire	Gustatory dysfunction	65.0	Speth et al. [46] *
Patients diagnosed by RT-PCR test	Mild to moderate	18 European hospitals (European 91.4%, African 4.6%, American 2.7%, Asian 0.8%)	1420	39.2	32.3	Self-reported questionnaire	Gustatory dysfunction	54.2	Lechien et al. [47]
OP and IP (diagnostic method, NS)	NS	USA	145	40	35.2	Self-reported questionnaire	Taste or smell change	65.5	Roland et al. [48]
HP (53.3%) and HQP (46.7%) diagnosed presumably by RT-PCR test	Asymptomatic (2.9%) Mild (48.7%) Moderate (40.6%) Severe (7.8%)	Italy	345	48.5 (23–88)	42.3	Objective evaluation using four tastants	Mild hypogeusia Moderate hypogeusia Severe hypogeusia Ageusia	22.6 7.2 4.6 10.4	Vaira et al. [49] *
Patients diagnosed by RT-PCR test	Mild to moderate	Belgium (Caucasian 92.9%, North African 7.1%)	28	44.0	32.1	Self-reported questionnaire based on taste and smell components of NHANES	Dysgeusia (salty, sweet, bitter, and sour tastes)	60.7	Lechien et al. [50] ^#^
HP diagnosed by RT-PCR test	NS	Germany	47	63.8	27.7	Self-reported questionnaire	Hypogeusia	19.1	Bertlich et al. [51]
OP diagnosed by real-time RT-PCR test	NS	France	95	39.8 (18–73)	16.8	Self-reported questionnaire	Dysgeusia	65.3	Zayet et al. [52]
OP (diagnostic method, NS)	Mild to moderate	Italy	95	NS	NS	Self-reported questionnaire	Gustatory dysfunction	50.5	De Maria et al. [53]
Patients diagnosed by RT-PCR test	NS	UK USA	Total: 7178 UK: 6452 USA: 726	UK: 41.3 USA: 44.7	UK: 28.1 USA: 21.9	Self-reported questionnaire via COVID-19 symptom tracker application	Taste and smell loss	Total: 65.0 UK: 64.8 USA: 67.5	Menni et al. [54]
Patients diagnosed by RT-PCR test	NS	Italy	355	50	54.1	Self-reported questionnaire	Gustatory dysfunction	65.4	Dell’Era et al. [55]
HP (diagnostic method, NS)	NS	Italy	103	55	57.3	Self-reported questionnaire	Dysgeusia	46.6	Liguori et al. [56]
Patients diagnosed by real-time RT-PCR test	NS	Turkey	64	37.8 (21–77)	39.1	Self-reported questionnaire	Dysgeusia Hypogeusia Ageusia	25.0 56.3 12.5	Sayin et al. [57]
IP and HQP diagnosed by real-time RT-PCR test	Mild to severe	Italy	IP: 295 HQP: 213	IP: 61.9 (24–91) HQP: 44.7 (18–74)	IP: 69.2 HQP: 38.0	Self-reported questionnaire	Gustatory dysfunction	IP: 51.9 HQP: 78.9	Paderno et al. [58]
Patients diagnosed by RT-PCR test	Mild to critical	Total Chinese 60.7% French 29.4% German 9.9%	394 239 116 39	38.8 30.5 48.1 43.1	57.1 58.4 59.6 48.1	Self-reported questionnaire	Gustatory dysfunction in Chinese French German	12.6 43.1 51.3	Qiu et al. [59] *
Household contacts of mildly symptomatic HQP (diagnostic test, NS)	NS	Italy	54	NS	NS	Self-reported questionnaire	Taste or smell loss	63.0	Boscolo-Rizzo et al. [60]
Patients diagnosed by real-time RT-PCR test	NS	USA	42	NS	NS	Self-reported questionnaire	Taste loss	57.1	Dawson et al. [61]
Patients diagnosed by RT-PCR test	NS	France	198	NS	NS	Self-reported questionnaire	Taste disorder	46.5	Tudrej et al. [62]
Patients positive for SARS-CoV-2 or quarantined patients (diagnostic test, NS)	NS	Spain	909	34 (16–74)	31.3	Self-reported online questionnaire	Gustatory dysfunction Ageusia Hypogeusia Dysgeusia	93.0 64.1 28.2 2.4	Gómez- Iglesias et al. [63]
Patients diagnosed by LT or CA (diagnostic tests, NS)	NS	Europe, North, Central and South America, Oceania, Middle East, Africa, Southeast Asia	Total: 4039 LT: 1402 CA: 2637	LT: 40.7 CA: 41.7	LT: 23.9 CA: 29.7	Self-reported questionnaire of changes in specific taste qualities	Single taste impairment Two or more taste qualities impairment	11 48	Parma et al. [64]
NHP (quarantined in hotel) diagnosed by RT-PCR test	Mild	Israel	128	36.3 (18–73)	45.3	Self-reported questionnaire	Gustatory dysfunction	52.3	Biadsee et al. [65] *
Patients (admitted to hospital or managed in community) diagnosed by real-time RT-PCR test	NS	UK	141	45.6 (20–93)	58.9	Self-reported questionnaire	Ageusia	63.1	Patel et al. [66]
OP (92.0%) and IP (8.0%) diagnosed by RT-PCR test	Mild to moderate	18 European hospitals (White 88.5%, South American 6.6%, North African 2.3%, Others 2.6%)	2013	39.5	34.0	Self-reported questionnaire based on taste and smell components of NHANES	Gustatory dysfunction	56.4	Lechien et al. [67]
Patients diagnosed by RT-PCR test	NS	Canada	134	57.1	47.8	Self-reported questionnaire	Dysgeusia	63.4	Carignan et al. [68]
IP (47.1%) and OP (52.9%) diagnosed by real-time RT-PCR test	Mild to critical	France	70	56.7 (19–96)	41.4	Self-reported questionnaire	Dysgeusia	48.6	Zayet et al. [69]
Patients diagnosed by RT-PCR test	NS	Spain	131	50.4	42.6	Self-reported questionnaire	Gustatory dysfunction Ageusia	56.5 42.7	Abalo-Lojo et al. [70] *
HP diagnosed by RT-PCR	NS	Italy (Caucasian)	100	65 (29–94)	60.0	Self-reported questionnaire	Gustatory dysfunction	69.0	Meini et al. [71]
HP diagnosed by real-time RT-PCR	Mild to critical	USA	50	59.6	58.0	Reviewed neurologic symptoms	Dysgeusia and hypogeusia	10.0	Pinna et al. [72]
NHP diagnosed by RT-PCR test	Mild to moderate	Poland	1942	50	39.8	Self-reported questionnaire	Gustatory dysfunction	47.5	Sierpiński et al. [73]
Patients diagnosed by RT-PCR test	Asymptomatic or oligosymptomatic	Poland	52	21.7 (19–26)	98.1	Self-reported questionnaire using four tastants	Taste or smell disturbance Sweet taste disorder	65.0 71.2	Adamczyk et al. [74] *^,#^
Patients diagnosed by RT-PCR test	NS	Latin Americans (Argentina 21.4%, Peru 17.2%, Colombia 15.5%, Uruguay 13.1%)	542	34 (18–88)	40.2	Self-reported online questionnaire	Gustatory dysfunction (salty, sweet, sour, and bitter tastes)	61.4	Chiesa- Estomba et al. [75]
OP diagnosed by RT-PCR test	NS	Canada	56	38.0	41.1	Self-reported symptoms	Dysgeusia/ageusia	57.1	Lee et al. [76]
Patients diagnosed by real-time RT-PCR test	NS	Iceland	1211	41.3–44.4	49.4–52.0	Self-reported symptoms	Taste or smell loss	9.7	Gudbjartsson et al. [77]
Discharged patients diagnosed by real-time RT-PCR test	Severe Non-severe	China	1172	61	49.2	Self-reported questionnaire	Ageusia	20.6	Song et al. [78]
HP (23.5%) and NHP (76.5%) diagnosed by RT-PCR test	NS	France	115	47 (20–83)	29.6	Self-reported questionnaire	Gustatory dysfunction	55.7	Chary et al. [79] *
Patients diagnosed by RT-PCR test	NS	European 88% Asian 12%	50	37.7 (18–65)	60	Self-reported questionnaire	Gustatory dysfunction	70.0	Freni et al. [80] *
OP with confirmed and suspected COVID-19 (diagnostic test, NS)	NS	Denmark	109	39.4	NS	Self-reported online questionnaire	Taste/smell loss Ageusia Hypogeusia	87.2 69.2 23.1	Fjaeldstad et al. [81]
HP diagnosed by real-time RT-PCR	NS	Turkey	81	54.2 (18–95)	50.6	Gustatory test using four tastants	Gustatory dysfunction	27.2	Altin et al. [82] *
IP (76.7%) and OP (23.3%) diagnosed by RT-PCR test	NS	Spain	846	56.8 (19–92)	52.7	Self-reported questionnaire	Taste loss in IP OP	48.4 65.0	Izquierdo- Dominguez et al. [83] *
OP diagnosed by RT-PCR test	Mild to moderate	Turkey	172	37.8 (18–65)	48.8	Self-reported questionnaire	Ageusia	47.1	Sakalli et al. [84]
HP or discharged patients diagnosed by real-time RT-PCR test	NS	Italy	204	52.6	53.9	Retrospective questionnaire	Taste loss	55.4	Mercante et al. [85] *
HP (diagnostic test, NS)	NS	Italy	108	59 (18–83)	57	Self-reported questionnaire	Gustatory dysfunction	61.1	Vacchiano et al. [86]
OP diagnosed by RT-PCR test	NS	Israel	73	(5–50)	NS	Self-reported questionnaire	Taste or smell impairment	50.7	Somekh et al. [87]
Healthcare personnel diagnosed real-time RT-PCR test	Mild	USA	51	NS	19	Self-reported questionnaire	Ageusia	52.9	Kempker et al. [88]
Healthcare workers seropositive for IgG antibodies against SARS-CoV-2	Mild to severe	Sweden	410	43	17.1	Self-reported questionnaire	Ageusia Anosmia	49.8 52.9	Rudberg et al. [89]
Healthcare stuff patients positive for COVID-19 (diagnostic test, NS)	NS	Italy	300	43.6 (33–53)	25.0	Self-scoring taste quality using four tastants	Ageusia Severe hypogeusia Moderate hypogeusia Mild hypogeusia	38.0 7.3 10.1 6.0	Petrocelli et al. [90] *
HP diagnosed by real-time RT-PCR test	Mild (98.8%)	China	86	25.5 (6–57)	51.2	Self-reported questionnaire	Hypogeusia	38.4	Liang et al. [91]
HP and HQP diagnosed by RT-PCR test	NS	Germany	41	37	32	Self-reported online questionnaire	Gustatory dysfunction	43.9	Hintschich et al. [92]
Patients tested by real-time RT-PCR test (92.7% with positivity)	NS	France	55	34 (28–43)	43.6	Self-reported chemosensory loss	Ageusia Dysgeusia	36.4 47.3	Salmon Ceron et al. [93] *
IP (41.0%) and OP diagnosed by RT-PCR test	Mild (59.0%) Moderate to severe (36.7%) Critical (4.3%)	France	139	48.5	37.4	Retrospective questionnaire one month after recovery	Ageusia	58.3	Poncet- Megemont et al. [94] *
Health care workers (nurse: 70.7%) diagnosed by RT-PCR test	NS	Italy	82	(<35–>55)	31.7	Self-reported questionnaire	Dysgeusia	37.8	Magnavita et al. [95]
OP (96.3%) and IP (3.7%) diagnosed by RT-PCR test or IgG/IgM antibodies test	Mild	European 79.6% Latin American 19.4%	1043	40 (18–78)	32.8	Self-reported questionnaire	Gustatory dysfunction	68.8	Chiesa- Estomba et al. [96]
Healthcare worker patients diagnosed by RT-PCR test	Mild to severe	Spain	230	43 (18–62)	14.8	Self-reported questionnaire	Taste alteration	70.0	Villarreal et al. [97] *
Patients diagnosed by real-time RT-PCR test	NS	Spain	215	NS	20.5	Self-reported questionnaire	Hypogeusia	53.0	Martin-Sanz et al. [98]
IP (23.2%) and OP (76.8%) diagnosed by RT-PCR test	NS	Italy	138	51.2	49.3	Self-administered test using four tastants	Gustatory dysfunction	65.9	Vaira et al. [99]
Patients diagnosed by real-time RT-PCR test	NS	Somalia	60	45.7	70.0	Retrospective symptom survey	Ageusia	28.3	Farah Yusuf Mohamud et al. [100]
OP diagnosed by RT-PCR test	NS	Spain	197	46.5 (21–89)	36.5	Self-reported questionnaire	Ageusia	65.0	Rojas-Lechuga et al. [101]
HP and NHP diagnosed by real-time RT-PCR test	Mild to critical	France	70	57	41.4	Retrospective symptom survey	Dysgeusia	48.6	Klopfenstein et al. [102]
IP (28.3%) and OP (71.7%) diagnosed presumably by RT-PCR test	NS	Italy	106	49.6	50.0	Self-administered gustatory test	Ageusia Hypogeusia	28.3 43.4	Vaira et al. [103]
HP (72.6%) and NHP (27.4%) diagnosed by RT-PCR test	Mild (18.4%) Moderate (61.4%) Severe (14.3%) Critical (5.8%)	Turkey	223	51	50.7	Self-reported questionnaire to score symptom severity	Taste loss	34.5	Salepci et al. [104] *
IP (16.8%) and OP (83.2%) diagnosed by RT-PCR test	Mild to critical	Brazil	655	37.7	35.3	Self-reported questionnaire	Gustatory dysfunction	76.2	Brandão Neto et al. [105]
Patients diagnosed by real-time RT-PCR test	Asymptomatic and mild to critical	Qatar	141	35.9 (3–56)	50.4	Retrospective symptom survey	Gustatory dysfunction Ageusia	19.9 11.4	Al-Ani et al. [106] *
Patients diagnosed by RT-PCR test	NS	Hong Kong	83	36.4 (18–71)	57.8	Self-reported questionnaire	Gustatory dysfunction	43.4	Cho et al. [107] *
HP diagnosed by RT-PCR test	Mild to moderate	Turkey	143	55.6	53.8	Self-reported questionnaire	Gustatory dysfunction	35.7	Çalıca Utku et al. [108] *
HP diagnosed by RT-PCR test	Manchester triage Green (53.5%) Yellow (30.2%)	Germany	43	71 (23–94)	65.1	Retrospective symptom survey	Dysgeusia	14.0	Fistera et al. [109]
HP diagnosed by real-time RT-PCR test, IgG/IgM antibodies test or both	Non-severe (60.9%) Severe (39.1%)	Spain	841	66.4	56.2	Retrospective symptom survey	Non-severe dysgeusia Severe dysgeusia	7.6 4.0	Romero- Sánchez et al. [110]
Patients diagnosed by RT-PCR test	NS	Japan	628	NS	53.7	Self-reported symptoms	Taste or smell loss	1.0	Komagamine et al. [111]
Patients diagnosed by real-time RT-PCR test	NS	Italy	111	57 (48–67)	52.3	Self-reported questionnaire	Dysgeusia	59.5	Fantozzi et al. [112] *
OP (94.6%) and IP (5.4%) diagnosed by RT-PCR test	Mostly mild	Israel	112	35	64.3	Self-reported questionnaire	Taste change as 1st symptom as ≥2nd symptom	25 54	Klein et al. [113] ^#^
Patients diagnosed by RT-PCR test	NS	USA (White 40.5%, Non-white 59.5%)	368	(<40–60+)	45.4	Self-reported questionnaire	Ageusia	28.5	Dixon et al. [114] ^#^

* indicates studies that were used to characterize symptom prevalence in discussion. # indicates preprint studies that precede formal peer review and publication (as of 28 February 2021). HP: hospitalized patients; NS: not specified; HQP: home-quarantined patients; OP: outpatients; IP: inpatients; NHP: non-hospitalized patients; LT: laboratory objective test; CA: clinical observational assessment; UPSIT: University of Pennsylvania Smell Identification Test; NHANES: National Health and Nutrition Examination Survey.

The retrieved 91 articles showed that the occurrence of gustatory dysfunction varies from study to study with the prevalence ranging from 1.0% to 93.0%, being almost consistent with the pooled prevalence of systematic reviews and meta-analyses reported thus far [115,116,117,118,119,120]. Only three systematic reviews have focused on gustatory dysfunction as follows. In an early review (from 1 January to 21 April 2020) of Aziz et al. [121], the prevalence of ageusia/dysgeusia was estimated to be 49.8% in five studies. Amorim dos Santos et al. [122] revealed that the prevalence of dysgeusia, hypogeusia, and ageusia are 38%, 35%, and 24%, respectively, in 33 studies between March and June 2020. The latest review of Cirillo et al. [123], including 67 eligible studies (27,687 COVID-19 cases, 16 countries, and multi-national cooperation), indicated that the overall reported prevalence of gustatory dysfunction shows geographical differences ranging from 5.6% to 96%. The prevalence and the characteristics of gustatory dysfunction associated with COVID-19 may depend on or relate to country or ethnicity, age, gender, and disease severity.

### 3.1. Country or Ethnicity

Early studies indicated that gustatory dysfunction is present in 70–90% of COVID-19 patients in Europe and USA, whereas in 5.6% in China [22,28]. Mao et al. [30] reported that the prevalence of taste impairment is 3.4% and 7.1% in severe and non-severe cases of patients admitted to hospital in Wuhan, China. In a study of Wong et al. [120], gustatory and olfactory dysfunctions were more frequently observed in European cohorts with the prevalence of 34–86%, in North American with 19–71%, and in Middle Eastern with 36–98% than in Asian cohorts with 11–15%. Taste loss and alteration are also less prevalent in South Korea and China than USA and Europe [117,124]. Even in Europe, the occurrence of gustatory and/or olfactory dysfunction is so dependent on country that the prevalence is 69.2% in Germany but 49.1% in France [59]. With respect to ethnicity, a systematic review and meta-analysis of von Bartheld et al. [118] revealed that gustatory dysfunction is six-fold more frequent in Caucasians than East Asians. Cirillo et al. [123] analyzed gustatory dysfunction of 67 studies including 16 countries. Their results indicated significant differences in the overall reported prevalence of gustatory dysfunction among countries (ranging from 11% of South Korea to 88.8% of Belgium), which may be due to genetic variation. SARS-CoV-2 uses ACE2 receptor for the entry into host cells and TMPRSS2 enzyme for the spike protein priming [125,126]. Genes encoding these proteins are variable depending on country or ethnicity [127]. A comparative genetic analysis of SARS-CoV-2 suggested that ACE2 and TMPRSS2 expression are different between different ethnics and between Asian and European populations [128,129]. There is also an ethnic difference in taste perception. Williams et al. [130] reported that Hispanics and African Americans rate taste sensations higher than non-Hispanic Whites.

### 3.2. Age

Children are considered less susceptible to SARS-CoV-2 compared with adults [131] and generally present with milder COVID-19 symptoms [132]. While neurological manifestations are relatively rare among children, gustatory dysfunction has been increasingly reported in younger cohorts [133]. Fontanet et al. [134] conducted a retrospective cohort study of primary school pupils aged six to 11 years in northern France and found that ageusia is present in only 1% of them. Taste and smell are significantly less impaired in Israeli children to show the prevalence of 25.8% compared with 71.4% in adults [87]. In contrast to these studies, Lee et al. [45] demonstrated that younger patients more frequently present with taste or smell loss in South Korea. Taste/smell disorder is more common in younger female patients in Spain [83], Italy [85], and Turkey [108]. Gustatory and olfactory dysfunctions were revealed to be present in 37.0% of 15- to 17-year-old children with mild and moderate disease [59]. Mak et al. [135] reported that children and adolescents infected with SARS-CoV-2 complain of ageusia and anosmia in the absence of other respiratory symptoms. In an objective evaluation of Vaira et al. [49], however, chemosensory dysfunction was not correlated with age, gender difference, and disease severity in Italian patients. Healthcare workers infected with SARS-CoV-2 developed taste and smell disorders independently of age [97].

### 3.3. Gender

Gustatory dysfunction associated with COVID-19 seems to depend on gender, as Chen et al. [9] and Giacomelli et al. [27] reported the prevalence of 52.6–57.1% in females but 25.0–36.5% in males. A European multicenter epidemiologic study indicated that females are more significantly affected by gustatory dysfunctions than males in mild to moderate cases [22]. When comparing the subjective symptoms, dysgeusia was present in 63.6% of females but in 33.9% of males in Italy [56]. Among non-hospitalized COVID-19 patients with milder disease, 52.8% of females and 39.6% of males developed taste disorders in Poland [73]. Ageusia and anosmia of females are more prevalent than those of males in South Korea [45], France [79], and Turkey [108]. A retrospective survey of Mercante et al. [85] showed that severe reduction of taste more frequently occurs in female patients. However, Levinson et al. [33] indicated that dysgeusia in mild cases is not associated with gender and age. There is no significant correlation between objectively assessed chemosensory dysfunction and gender or age [49], and the prevalence of taste and smell disorders is independent of gender in healthcare workers infected with SARS-CoV-2 [97]. Genotype tissue expression (GTEx) database shows that ACE2 and TMPRSS2 expression is not different between males and females [129,136]. Cai et al. [137] comparatively analyzed Caucasian and Asian RNA-seq data sets, but they found no significant relations between gender and ACE2 expression. Taste perception can vary as a function of gender [130]. Females show greater taste responsiveness than males [138], which is supported by anatomical data that females have more fungiform papillae and more taste buds than males [139]. Gender differences in taste perception may be associated with those in gustatory dysfunction.

### 3.4. Disease Severity

Early COVID-19 studies showed that taste is more frequently altered in Chinese non-severe patients [30] and in Italian mild outpatients [32] compared with severe cases. Such a relation to disease severity is consistent with the comparative results that inpatients were less likely to present with dysgeusia than outpatients [41]. In a cross-sectional study, gustatory dysfunction was present in 78.9% of home-quarantined (milder) patients but in 51.9% of hospitalized (severe/moderate) patients in Italy [58]. Taste or smell loss occurred in 79.6%, 14.8%, 3.5%, and 2.2% of COVID-19 patients with asymptomatic to mild, moderate, severe, and critical disease, respectively, in South Korea [45]. Qiu et al. [59] demonstrated that the prevalences of gustatory and olfactory dysfunction are 48.4%, 24.8%, 20.5% and 6.2% in mild, moderate, severe, and critical COVID-19 cases, respectively. When comparing disease severity of Turkish patients, taste loss was more common in mild to moderate cases to show the prevalence of 36.5% compared with severe to critical cases to show the prevalence of 26.7% [104]. Ageusia was most prevalent in mildly symptomatic patients, followed by moderate and severe patients in Qatar [106]. Borsetto et al. [140] systematically analyzed self-reported taste or smell alteration and revealed that the prevalence is 47% overall, while it is 67% and 31% in mild to moderate and severe patients. Gustatory dysfunction is associated with relatively mild severity of COVID-19.

### 3.5. Assessment Method

Gustatory function is exclusively assessed by subjective or self-reported impressions of COVID-19 patients on taste alteration, whereas a small number of objective studies were performed by applying primary tastants to the tongue [22,49,74,82,90]. Although taste sensitivity is reduced with increasing age and is higher in females than males, the objective test has been considered reliable to assess the tasting ability [141]. Vaira et al. [42] compared self-administered and operator-administered tests using solutions of salt, sugar, lemon juice, and decaffeinated coffee. Their results indicated that the prevalence of gustatory dysfunction is not different between two tests. A comparative analysis of von Bartheld et al. [118] revealed that a difference between subjective and objective tests of chemosensory dysfunction does not reach significance.

### 3.6. Quantitative Dysfunction

Gustatory dysfunction includes ageusia (complete taste loss) and dysgeusia (taste impairment). Dysgeusia is classified into mild hypogeusia (or amblygeusia), moderate hypogeusia, and severe hypogeusia. A cross-sectional study indicated that the prevalence is 8.5% for dysgeusia and 1.7% for ageusia in Italian COVID-19 patients [27]. Vaira LA et al. [31] demonstrated that mild hypogeusia, moderate hypogeusia, severe hypogeusia, and ageusia are present in 22.2%, 15.3%, 9.7%, and 1.4% of Italian COVID-19 patients, respectively. Their following study showed that gustatory dysfunction of home-quarantined patients is composed of mild hypogeusia (the prevalence of 24.2%), moderate hypogeusia (12.1%), severe hypogeusia (9.1%), and ageusia (6.1%) [42]. In a multicenter cohort study, dysgeusia and ageusia occurred in 47.3% and 36.4% of French COVID-19 patients [93]. In contrast to these studies, Spinato et al. [32] reported that Italian outpatients complain of severe, moderate, mild, and very mild gustatory dysfunction in order of descending frequency. Beltrán-Corbellini et al. [38] also demonstrated that the prevalences of ageusia, dysgeusia, and hypogeusia are 45.2%, 25.8%, and 22.6%, respectively, of Spanish patients. Severe, moderate, and mild gustatory dysfunction were present in 67.2%, 22.4%, and 10.4%, respectively, in Switzerland [46]. Among Turkish patients reporting ageusia, 30.2%, 10.5%, and 6.4% were severe, moderate, and mild symptoms, respectively [84]. Petrocelli et al. [90] characterized gustatory dysfunction at an early stage of COVID-19 and indicated that the prevalences of ageusia, severe hypogeusia, moderate hypogeusia, and mild hypogeusia are 38.0%, 7.3%, 10.1%, and 6.0%, respectively. In two multicenter cross-sectional studies of Spanish COVID-19 patients [83,101], severe, moderate, and mild taste loss were present in 52.9–65.6%, 28.1–37.1%, and 6.3–10.0%, respectively. Taste loss of Brazilian patients was composed of severe, moderate and mild symptom to show the prevalences of 45.5%, 23.4%, and 7.8%, respectively [105]. In Hong Kong, 26.5% and 8.4% of COVID-19 patients presented with severe hypogeusia and mild to moderate hypogeusia [107].

### 3.7. Qualitative Dysfunction

Taste cells express one of the taste receptors that specifically interacts with salty, sweet, sour, bitter, and umami tastant to transfer the taste information through taste nerve fibers to the central nervous system (CNS) [142]. However, many studies thus far overlooked or poorly understood the qualitative alteration of individual tastes. Symptomatology of European and Asian COVID-19 patients indicates that gustatory dysfunction includes impairments of salty, sweet, sour, and bitter tastes [80]. When characterizing gustatory dysfunction of COVID-19 patients, the prevalence of sweet, salty, and sour taste loss showed the prevalences of 47.7%, 42.2%, and 41.4%, respectively [65]. Abalo-Lojo et al. [70] demonstrated that complete taste, bitter taste, salty taste, and sweet taste loss are present in 75.7%, 8.1%, 4.1%, and 1.4% of COVID-19 patients, respectively. Adamczyk et al. [74] qualitatively assessed gustatory disorder by using solutions of sucrose (40–106.4 mg/mL), NaCl (13.5–27 mg/mL), ascorbic acid (6.25–12.5 mg/mL), and grapefruit extract (40 mg/mL). Sweet taste was especially impaired in COVID-19 patients, who could not recognize the lowest sucrose concentration. Parma et al. [64] analyzed the results of international questionnaires of laboratory objective test groups and clinical observational assessment groups. Among all the tested and assessed COVID-19 patients, 48% and 11% reported impairments of two or more tastes and of a single taste. With respect to a specific taste quality, salty, sweet, bitter, sour, and umami tastes were impaired in 44.9%, 44.8%, 39.2%, 37.9%, and 29.3% of laboratory-tested patients, respectively, and in 45.9%, 44.0%, 39.3%, 37.2%, and 25.3% of clinically assessed patients, respectively. Differential characterization of ageusia and dysgeusia indicated that the recognition failure of COVID-19 patients is most prevalent in salty taste, followed by sour, bitter, and sweet taste [93]. Qualitative gustatory dysfunction may give insights into the pathogenic mechanisms as discussed in Section 4.1 and Section 4.2.

### 3.8. Onset

Gustatory dysfunction precedes the onset of full-blown disease [27] or occurs at an early stage of SARS-CoV-2 infection and in asymptomatic or paucisymptomatic patients [23,30]. A clinical course analysis indicated that COVID-19 patients develop ageusia and anosmia within the first 5 days of the clinical onset [31]. Abalo-Lojo et al. [70] demonstrated that 84.8% of gustatory and olfactory dysfunctions appear in the first 4 days. Levinson et al. [33] determined the median onset of taste and smell alteration to be 3.3 days. In a multicenter prospective study, ageusia or severe hypogeusia was present in 40.6% of Italian patients within the first 4 days of COVID-19 symptom onset [99]. A retrospective cohort study showed that 78.8% of patients report taste disorder before the COVID-19 diagnosis [112]. Gustatory dysfunction is considered as an early symptom of COVID-19 [22,143].

### 3.9. Duration and Recovery

Levinson et al. [33] reported that the median duration of dysgeusia associated with COVID-19 is 7.1 days. A comparative study of Italian patients indicated that the mean duration of gustatory dysfunction is 16 days, and the mean recovery time from gustatory dysfunction is 26 days in females, longer than the 14 days in males [71]. Liang et al. [91] revealed that the duration of hypogeusia is 7.1 days in Chinese patients. The average duration of gustatory and olfactory disorders was found to be 11 days in healthcare workers infected with SARS-CoV-2 [97]. Salepci et al. [104] characterized that the median duration time of gustatory dysfunction is 11 days in severe to critical cases but 7 days in mild to moderate cases. The majority of South Korean patients recovered from ageusia within three weeks with the median resolution time of 7 days [45]. Al-Ani and Acharya [106] demonstrated that COVID-19 patients in Qatar fully recover from ageusia and anosmia in a mean of 6.9 days, ranging from 3 to 12 days. A prospective cross-sectional study of Hong Kong cohort showed that the mean recovery time of gustatory dysfunction is 9.5 days [107].

In European and Asian cohorts, gustatory dysfunction remained in 8% of cases after the resolution of COVID-19 symptoms or after 15 days from the RT-PCR test negativity [80]. Although 50% of COVID-19 patients fully recovered from gustatory dysfunction in Denmark, 20% did not experience any improvement at a mean of >30 days after taste loss [81]. Even if one month passed after the disappearance of fever and dyspnea, ageusia still persisted in 11.5% of French patients [94]. In a study of European multiple sites, 9.4% of patients reported gustatory dysfunction after a mean follow-up of 63 days from the first consultation [96]. A longitudinal study showed that taste alteration of Israeli patients lasts long with the mean duration of 17.2 days [113]. Boscolo-Rizzo et al. [144] followed up symptoms of mildly symptomatic COVID-19 patients from baseline to four and eight weeks. The prevalence of taste/smell impairment was 60.1% for baseline, 36.6% for four weeks, and 18.6% for eight weeks. Carfi et al. [145] conducted a follow-up study of COVID-19 patients discharged from hospital and revealed that symptoms including taste alteration persist for a mean of 60.3 days after the onset of COVID-19 symptoms. In mild to moderate cases, gustatory dysfunction, ageusia, and hypogeusia persisted in 21.5%, 11.6%, and 9.9% of COVID-19 patients, respectively, at a mean follow up time of 38.2 days [146]. Cirulli et al. [147] analyzed self-reported long-term symptoms in adult populations and demonstrated that 36.1% of mild COVID-19 patients present with symptoms such as ageusia lasting longer than 30 days. Gustatory dysfunction is considered to persist in many patients discharged from hospital.

### 3.10. Association with Olfactory Dysfunction

The prevalence and the risk factor of gustatory dysfunction overlap with those of olfactory dysfunction, as suggested by COVID-19 epidemiologic studies [22,44,46,93]. Klopfenstein et al. [37] reported that dysgeusia and anosmia co-occur in 85% of COVID-19 patients. Altin et al. [82] demonstrated that the prevalence of dysgeusia without anosmia is only 1.2%, indicating a close relation between taste and smell alteration. In COVID-19 symptomatology, severity of gustatory dysfunction is strongly correlated with that of olfactory dysfunction [46]. An age-matched case-control study demonstrated that 50% of Canadian patients present with both dysgeusia and anosmia but 13.4% with only dysgeusia [68]. A retrospective study also revealed that 92.6% of French patients develop ageusia together with anosmia [94]. A positive association between gustatory and olfactory dysfunction in COVID-19 may suggest the pathogenic mechanism common to both symptoms.

## 4. Possible Mechanisms of Gustatory Dysfunction

Although nasal congestion is a risk factor of taste and smell problems [148], it is not directly related to gustatory dysfunction associated with COVID-19 patients because a substantial number of patients with taste impairment do not develop nasal congestion [61,63,79]. Taste alteration of COVID-19 patients is not necessarily accompanied by nasal obstruction and rhinitis [23,26].

### 4.1. ACE2 Expression in Taste Cells

By exploring public genomic databases, Xu et al. [4] showed that mucosal epithelial cells of the oral cavity express ACE2 receptors for SARS-CoV-2. They also confirmed that ACE2 is expressed in human oral tissues, especially enriched in dorsal tongue more than gingival and buccal tissues. Sato et al. [149] found that ACE2 and TMPRSS2 responsible for the viral cellular entry are co-expressed in the taste buds of tongue foliate papillae of rats. Wang et al. [150] revealed that ACE2 is enriched in a subpopulation of mouse tongue epithelial cells. Immunohistochemical experiments of Sakaguchi et al. [5] indicated that ACE2 and TMPRSS2 are consistently expressed and localized in human fungiform papillae taste cells.

Han et al. [151] analyzed the single cell profiles of tongue tissues with typical gene markers of taste buds such as TAS1R3, TAS2R4, TAS2R14, SNAP25, and NCAM1. They found that the distribution of ACE2-positive cells is correlated with that of taste-relating gene marked cells. In subpopulations of taste bud cells, type II cells respond to sweet, umami, and bitter stimuli, and type III cells to sour stimuli and high concentration salt [152]. Type II marker genes include TAS1R2 and TAS1R3 for sweet taste, TAS1R1 and TAS1R3 for umami taste, and TAS2Rs for bitter taste, and type III marker genes include NCAM1 and SNAP25 [153]. The distribution of type II and type III marker genes in the tongue suggests the co-existence of ACE2 and individual taste cells, being consistent with impairments of sweet, bitter, and umami tastes and of salty and sour tastes in COVID-19 patients. SARS-CoV-2 would directly damage ACE2-expressing cells of taste buds during the cellular entering process, resulting in gustatory dysfunction [154].

A series of studies of Shigemura et al. showed that angiotensin II suppresses amiloride-sensitive taste responses to NaCl and enhances nerve responses to sweeteners [155] and that three renin-angiotensin system (RAS) components are present in taste buds of fungiform and circumvallate papillae [156]. These results suggest that angiotensin II and ACE2 play a critical role in taste perception and modulation because the RAS produces angiotensin II and ACE2 degrades angiotensin II to angiotensin-(1-7). This is supported by a study of Tsuruoka et al. [157] that angiotensin II receptor blocker losartan and ACE inhibitor perindopril attenuated sweet, salty, sour, and bitter tastes of volunteers. Kuba et al. [158] reported that SARS-CoV infection reduces the expression of ACE2 in mouse lungs. Yang et al. [159] also showed that ACE2 expressions in Vero E6 cells and mouse lungs infected with SARS-CoV-2 significantly decrease compared with control groups. While cytokine storm underlies the severe symptoms of COVID-19, interleukin 7 (IL-7) and IL-2 increase in plasma of COVID-19 patients [3], and IL-7 and IL-2 reduce the expression of ACE2 in mice [160]. SARS-CoV-2 infection may modify the activity of ACE2 with the subsequent change of angiotensin II levels in taste buds, resulting in gustatory dysfunction.

### 4.2. Zinc Deficiency

Zinc is an element essential for not only the immune system and the inflammatory response but also the gustatory function at a level of taste buds and taste stimulus-transmitting nerves [161]. Zinc is also required for regeneration and maintenance of taste cells and for zinc-metalloenzymes localized in taste buds [162]. Taste disorders are improved by administrating zinc [163]. Zinc ionophore chloroquine to increase the cellular influx of zinc was found to inhibit SARS-CoV-2 in vitro [164].

Jothimani et al. [165] determined zinc concentrations in serum of COVID-19 patients at the time of hospitalization. The patients showed significantly lower concentrations compared with healthy controls. When zinc level <80 μg/dL was defined as deficiency, 57.4% of patients were deficient in zinc, and they had higher rates of complications, prolonged hospitalization, and increased mortality. Vogel-González et al. [166] also measured serum zinc of COVID-19 patients admitted to the hospital. Serum zinc levels lower than 50 μg/dL at admission correlated to worse clinical presentation, longer time to reach stability, and higher mortality. Their Vero E6 cell culture experiment indicated that low zinc levels are favorable to the expansion of SARS-CoV-2 in cells. Okayama and Watanabe [167] comparatively evaluated nutrient intake and taste perception of 74 women in their late teens and twenties. When assessing taste alteration by using filter-paper discs of four tastants, 44.6% of subjects showed insensitive taste, which was associated with lower zinc intake. Among such subjects, 29.7% presented with one abnormal taste (sweet, salty, sour, or bitter taste) and 10.8% with two abnormal tastes (sweet and another taste or salty and bitter taste). When rats were fed zinc-deficient diets, taste gene marker TAS2R-positive cells and epithelial sodium channel-positive cells were decreased in vallate taste buds [168]. These results suggest that zinc deficiency may induce impairments of sweet, salty, and bitter tastes, being consistent with the qualitative gustatory dysfunction of COVID-19 patients. A decreased zinc level is also favorable for the interaction of zinc-dependent metalloenzyme ACE2 with spike proteins of SARS-CoV-2 but an increased zinc level inhibits ACE2 expression [165]. Takeda et al. [169] compared serum zinc concentrations of zinc-deficient taste impairment patients, idiopathic taste impairment patients, and controls. The mean concentrations were 77.4 μg/dL for controls and 77.6 μg/dL for idiopathic patients but 55.7 μg/dL for zinc-deficient patients. Although there was no significant difference in serum ACE activity among these groups, the apo/holo ACE activity ratio increased to 13.7 in zinc-deficient patients and 9.8 in idiopathic patients compared with 1.10 in controls. Zinc deficiency is considered as one of causative factors for gustatory dysfunction associated with COVID-19, even if zinc levels are seemingly within normal ones.

### 4.3. Hyposalivation

Saliva functions as a solvent of taste substances and regulates the conditions of taste receptors, therefore, a change of saliva flow is potentially linked to taste alteration of COVID-19 patients [170]. A clinical case that the treatment of dry mouth improved hypogeusia in the elderly indicates a relation between hyposalivation and gustatory dysfunction [171]. Taste disorders involve the damage of gustatory papillae and taste buds caused by reduced saliva secretion [162]. COVID-19 patients frequently develop xerostomia as described in Section 5. Biadsee et al. [65] reported that the occurrence of xerostomia is correlated with that of gustatory dysfunction in COVID-19. Since human salivary glands express ACE2 and TMPRSS2, salivary gland infection with SARS-CoV-2 would affect saliva secretion, thereby altering the tasting ability. Zinc increases the secretion of unstimulated and stimulated whole saliva in humans [172]. Conversely, zinc deficiency associated with COVID-19 may induce gustatory dysfunction through hyposalivation.

### 4.4. Taste Cell Inflammation

Since taste cells express higher levels of several inflammatory receptors and signaling proteins, Cohn et al. [173] investigated the effects of lipopolysaccharide-induced inflammation on taste progenitor cells and taste bud cells of mice. They found that the inflammation attenuates cell proliferation and interferes with taste cell renewal, suggesting that inflammation may be related to taste disorders associated with infectious diseases. Wang et al. [150] speculated that SARS-CoV-2 infection possibly causes ageusia by the virus-induced inflammation and the release of pro-inflammatory cytokines. Taste bud cells express cytokine signaling pathways through which the inflammation would affect gustatory function [174]. Inflammatory cytokines also trigger apoptotic cell death to cause the abnormal turnover in taste buds, leading to gustatory dysfunction.

### 4.5. Viral Neuroinvasion

Neurological manifestations of COVID-19 are attributed to disturbance of the peripheral nervous system (PNS) and/or the CNS [175]. Since SARS-CoV-2 targets nerves of the PNS, the direct damage to any of cranial nerves involved in taste stimulus transmission is presumed to induce gustatory dysfunction. The facial nerve (cranial nerve VII) and the glossopharyngeal nerve (cranial nerve IX) innervate the anterior two-thirds of tongue and the posterior one-third of tongue. The epiglottis and the taste buds are innervated by the internal laryngeal branch of the superior laryngeal nerve, which is a branch of the vagus nerve (cranial nerve X), to carry the taste information to the CNS. Among these nerves, the cranial nerve VII damaged by SARS-CoV-2 may be primarily responsible for gustatory dysfunction in COVID-19 [13].

Hoang et al. [176] proposed a pathogenic mechanism that SARS-CoV-2 enters oral epithelia through ACE2 and TMPRSS2 to damage the taste receptors and infiltrate the CNS. Keyhan et al. [177] also hypothesized that SARS-CoV-2 may degrade the CNS by stimulating T cell-mediated autoimmune reactions to CNS antigens, thereby disturbing gustatory function. Since meningitis and encephalitis last longer and are less prevalent than dysgeusia, involvement of the CNS seems less likely compared with the PNS.

## 5. Xerostomia

Results of the literature search indicated that saliva secretion is frequently affected by SARS-CoV-2 infection. Table 2 summarizes the studies of xerostomia associated with COVID-19.

### 5.1. Prevalence and Symptom Characterization

Chen et al. [9] first referred to xerostomia in COVID-19 patients. In their study, dry mouth was present in 46.3% overall, 22.2% of males and 24.1% of females. The prevalence of xerostomia associated with COVID-19 seems unlikely to vary by country, as it ranged from 46% to 56% in Italy, China, and Israel [9,65,112], although the number of relevant studies and their sample sizes are small. In a web-based questionnaire study, xerostomia was reported by 21.9% of males and 34.4% of females [65], which may suggest the prevalence depending on gender. Niklander et al. [178] examined the oral health of dental patients and revealed that xerostomia more frequently occurs in females and with increasing age. Freni et al. [80] determined the prevalence of xerostomia in European and Asian cohorts during the active phase of COVID-19 symptoms and 15 days after the RT-PCR test negativity. In the former condition, 32.0% of patients presented with xerostomia, which persisted in 2.0% of subjects at the disappearance of COVID-19 symptoms. In a retrospective study, 74.5% of patients with the oral symptoms reported xerostomia before the diagnosis of COVID-19 [112].

### 5.2. Clinical Significance

A follow-up study suggested that hyposalivation may be a risk factor of acute respiratory infection in dental outpatients [179]. Hyposalivation exposes people to a higher risk of SARS-CoV-2 infection by disturbing the oral mucosal surface as a physical barrier against viruses and decreasing the secretion of antiviral proteins and peptides in saliva [180]. In addition, reduction of saliva secretion is linked to gustatory dysfunction as speculated in Section 4.3. Xerostomia is usually secondary to nasal congestion and results from mouth breathing. However, there is no significant correlation between xerostomia and nasal congestion of COVID-19 patients [65].

## 6. Possible Mechanisms of Xerostomia

### 6.1. ACE2 Expression in Salivary Glands

ACE2 and TMPRSS2 responsible for the cellular entry of SARS-CoV-2 are expressed in human salivary glands [4,10], and they are also distributed in submandibular, parotid, and minor salivary glands of mice [6]. Chen et al. [160] analyzed GTEx database and other public data of 30 tissues across thousands of individuals. Consequently, they revealed that ACE2 is more highly expressed in salivary glands of Asian females compared with Caucasian and African people. SARS-CoV-2 targets ACE2-localizing salivary glands, damages them, and affects their secretion function, resulting in xerostomia or saliva flow reduction. Hyposalivation is also linked to gustatory dysfunction as speculated in Section 4.3. Interestingly, a pairwise correlation analysis of Luo et al. [181] indicated a correlation between dry mouth and bitter taste of COVID-19 patients.

### 6.2. Inflammation of Salivary Glands

Clinical cases of inflamed salivary glands have been reported for COVID-19 patients as described in Section 7.2. Based on the expression and the distribution of ACE2 in salivary glands, Wang et al. [16] proposed the following hypothesis. SARS-CoV-2 could cause acute sialadenitis by binding to ACE2 receptors in the epithelia of salivary glands and subsequently lysing the cells. Although the inflammatory damages of acinar cells are repaired by fibroblast proliferation and fibrous connective tissue formation, fibrous repair and hyperplasia may cause salivary gland hyposecretion and stenosis of the ducts of salivary glands, thereby reducing saliva flow.

### 6.3. Zinc Deficiency

Kim et al. [172] collected unstimulated and stimulated whole saliva from patients with hyposalivation, Sjögren syndrome patients, and healthy subjects. Mouth rinsing with 0.25% ZnCl_2_ solution increased both saliva in all groups, indicating that zinc stimulates saliva secretion. They also found the expression in human salivary glands of metabotropic zinc receptor/G-protein-coupled receptor (ZnR/GPR39) that modulates saliva secretion from submandibular glands. Since many COVID-19 patients show lower serum zinc concentrations [165,166], zinc deficiency may contribute to xerostomia associated with COVID-19.

### 6.4. Viral Neuroinvasion

Parotid and submandibular glands are innervated by autonomic and sensory nervous systems. SARS-CoV-2 enters peripheral nerves through the trans-synaptic pathways [80]. The virus may damage the PNS during their neuroinvasion, affecting the function of salivary glands.

## 7. Other Oral Symptoms

Oral symptoms other than gustatory dysfunction and xerostomia have been suggested for COVID-19, although it is still unclear whether they are due to SARS-CoV-2 infection or secondary manifestations. Almost all of them are case reports as short communications or letters to the editor.

### 7.1. Mucosal Lesions

The relevant reports [17,182,183,184,185,186,187,188,189,190,191,192,193] include 13 male cases (age: 24–81 years) and 10 female cases (age: 32–83 years). COVID-19 patients developed ulcers on hard palate, tongue and buccal mucosa, erosions of lips and buccal mucosa, multiple reddish macules of hard palate, tongue and lips, blisters of labial mucosa, stomatitis aphthous, geographic tongue, desquamative gingivitis, angular cheilitis, etc. Some patients had the history of diabetes, hypertension, obesity, coronary heart disease, kidney disease, and/or kidney transplant, which had been subjected to treatments with immunosuppressants, diuretics, or ACE inhibitors. Iranmanesh et al. [194] published an excellent paper to review 35 articles on oral manifestations of COVID-19 and gave important information as follows. The most common site of lesions is tongue (38%), followed by labial mucosa (26%) and palate (22%). The prevalence of oral lesions is nearly equal in both genders (49% female and 51% male). Patients with older age and higher disease severity develop more widespread and severe oral lesions. They suggested that lack of oral hygiene, opportunistic infections, stress, immunosuppression, vasculitis, and hyper-inflammatory response secondary to the viral infection are linked to oral mucosal lesions associated with COVID-19. SARS-CoV-2 infection could aggravate oral pathological conditions of patients, especially those with compromised immune system and long-term pharmacotherapy.

### 7.2. Sialadenitis

SARS-CoV-2 could cause sialadenitis of submandibular salivary glands and inflammation of parotid salivary glands (parotitis). In two cases of Chern et al. [195], computed tomography imaging indicated enlarged parotid and submandibular glands of an 88-year-old woman and a 64-year-old man. Lechien et al. [196] reported parotitis-like symptoms associated with COVID-19 in 23-, 27- and 31-year-old women who had taste and smell loss. In a case report of Capaccio et al. [197], a 26-year-old man developed left painful parotid swelling, the ultrasonography of which showed enlarged and diffuse hypoechoic parotid gland structure.

### 7.3. Periodontal Disease

ACE2, TMPRSS2, and furin play an important role in the viral cell invasion and are expressed in sulcular and periodontal pocket epithelium [5]. Therefore, SARS-CoV-2 potentially infects these epithelia and adversely influences periodontal tissues. Since major periodontopathic bacteria *Prevotella intermedia* is frequently detected in COVID-19 patients, SARS-CoV-2 could predispose individuals to a periodontal disease through bacterial co-infection propagated by *Prevotella intermedia*. Patel and Woolley [18] reported one case that a 35-year-old woman with suspected COVID-19 developed halitosis, intense gingival pain, and bleeding from the gingival sulcus. Her intraoral examination showed severe halitosis, generalized erythematous and edematous gingivae, and necrotic interdental papillae in both maxillary and mandibular labial sextants. Riad et al. [198] reported a series of 18 cases of COVID-19 patients (4 males and 14 females, age ranging from 18 to 72 years) who commonly complained of halitosis.

## 8. Dental Implications

Gustatory and olfactory symptoms predict COVID-19 with the sensitivity of 70% and the specificity of 73–90.3% [48,93]. When assessing COVID-19 patients by using solutions of sucrose (106.4 mg/mL) and NaCl (13.5 or 17 mg/mL), ageusia of sweet and/or salty taste corresponds to the highest accuracy diagnostic test with the specificity of 100% and the sensitivity of 34% [74]. Given the onset time and the relation to disease severity, gustatory dysfunction is usable as a symptom for COVID-19 diagnosis at an early stage and in asymptomatic patients. Xerostomia is also valuable to detection and diagnosis of COVID-19 as well as gustatory dysfunction because both oral symptoms have the relatively early onset and appear prior to other symptoms.

Saliva has different effects on COVID-19. Salivary glands infected with SARS-CoV-2 secrete the virus-contaminated saliva that is primarily responsible for human-to-human transmission. Since saliva droplets and aerosols are routinely generated during dental procedures, not only patients have a risk of exposure to SARS-CoV-2 during dental treatments but also dentists and dental hygienists constantly confront the highest risk of SARS-CoV-2 infection [199]. Dental professionals and oral healthcare workers must make as much effort as possible in pre-, intra-, and peri-operative prevention of SARS-CoV-2 infection [200]. On the other hand, the virally infected saliva is usable for COVID-19 diagnosis because a saliva specimen can be easily collected without any invasive procedures to patients and high-risk exposure to collectors. The SARS-CoV-2 viral load in saliva and the SARS-CoV-2 detection sensitivity of self-collected saliva are almost comparable to nasopharyngeal swabs [201,202]. The use of saliva facilitates wider PCR testing.

## 9. Conclusions

The present review demonstrates that the oral cavity is one of the most vulnerable areas to SARS-CoV-2. Gustatory dysfunction and xerostomia are possibly the symptoms presenting before other COVID-19 manifestations and the subjective complaints in asymptomatic or mild COVID-19 cases, and they persist relatively long, while the prevalence depends on country or ethnicity, age, gender, and disease severity. Oral mucosal lesions are also suspected to be caused by SARS-CoV-2 infection. Gustatory dysfunction, xerostomia, and mucosal ulceration associated with COVID-19 could negatively affect oral health, and poor oral hygiene of the hospitalized COVID-19 patients could adversely affect these oral symptoms.

The oral symptoms specific to COVID-19 patients enhance the relevance of dentistry to COVID-19. Dentists and dental hygienists who can first become aware of taste alteration, dry mouth, and mucosal lesions should pay close attention to such symptoms and perform an exhaustive intraoral examination to identify COVID-19 patients at an early stage. By understanding the oral symptoms, dental professionals will play a more active role in and make a greater contribution to management of COVID-19 as suggested by Ren et al. [203].

There are no recognized pathogenic mechanisms thus far to explain gustatory dysfunction and xerostomia of COVID-19 patients. Further studies are necessary to elucidate them in order to better understand the features of SARS-CoV-2 infection and to get new insights into diagnosis and preventive measures of COVID-19. The present mechanistic speculation would be helpful for such a research subject.

## Figures and Tables

**Table 2:** Xerostomia associated with coronavirus disease 2019 (COVID-19).

Subjects	Severity of Disease	Country or Ethnicity	Number of Patients	Mean or Median Age (Range)	Male (%)	Assessment Method	Symptom	Prevalence (%)	Reference
Hospitalized patients diagnosed according to the official guideline and by SARS-CoV-2 nucleic acid detection	Mild to critical	China	108	52.0 (one patient excluded)	48.1	Self-reported questionnaire	Dry mouth	46.3	Chen et al. [9] *
Ambulatory non-hospitalized patients (quarantined in hotel) diagnosed by RT-PCR test	Mild	Israel	128	36.3 (18–73)	45.3	Self-reported web-based questionnaire	Xerostomia	56.3	Biadsee et al. [65] *
Patients diagnosed by RT-PCR test	NS	European 88% Asian 12%	50	37.7 (18–65)	60.0	Self-reported questionnaire	Xerostomia	32.0	Freni et al. [80] *
Patients diagnosed by real-time RT-PCR test	NS	Italy	111	57 (48–67)	52.3	Self-reported questionnaire to rate xerostomia scores	Xerostomia	45.9	Fantozzi et al. [112] *

* indicates studies that were used to characterize symptom prevalence in discussion. NS: not specified.
